# Supplementary material for: Characterization of regulatory transcriptional mechanisms in hepatocyte lipotoxicity
Source: Sci Rep. 2022 Jul 7;12:11477. doi: 10.1038/s41598-022-15731-4 (PMC9262951; doi:10.1038/s41598-022-15731-4)
Supplement: Supplementary file 1 — Supplementary Information. [file 41598_2022_15731_MOESM1_ESM.pdf]

Characterization of regulatory transcriptional mechanisms in hepatocyte lipotoxicity

Joaquín Pérez-Schindler[1,2,*], Elyzabeth Vargas-Fernández[1], Bettina Karrer-Cardel[1], Danilo Ritz[1], Alexander Schmidt[1], Christoph Handschin[1,*]

[1]Biozentrum, University of Basel, Basel, 4056, Switzerland.

[2]Present address: Broad Institute of MIT and Harvard, Cambridge, MA 02142, USA.

*Corresponding authors: jperezsc@broadinstitute.org; christoph.handschin@unibas.ch

Supplementary tables

Table_S1_RNAseq_NASH_and_PAL

Table_S2_Metascape_RNAseq_GO_analysis

Table_S3_Proteomics_NASH_and_PAL

Table_S4_Motif_Enrichment_Analysis _NASH_and_PAL

Table_S5_ISMARA _TF_activity_NASH_and_PAL

Table_S6_RNAseq_TCF4_KO

Table_S7_RNAseq_MAFK_KO

Table_S8_RNAseq_ATACseq_sequencing_summary

Supplementary figures

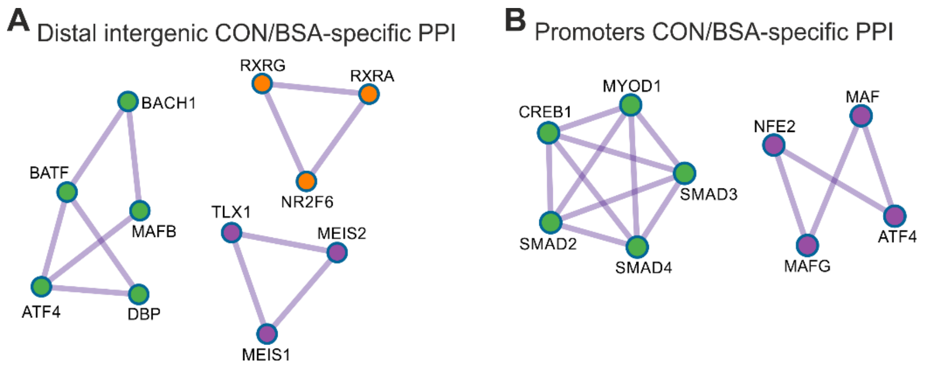

Figure S1. **Protein-protein interaction (PPI) network analysis.** (A and B) PPI networks of CON/BSA-specific transcription factors at (A) distal intergenic regions and (B) promoters.

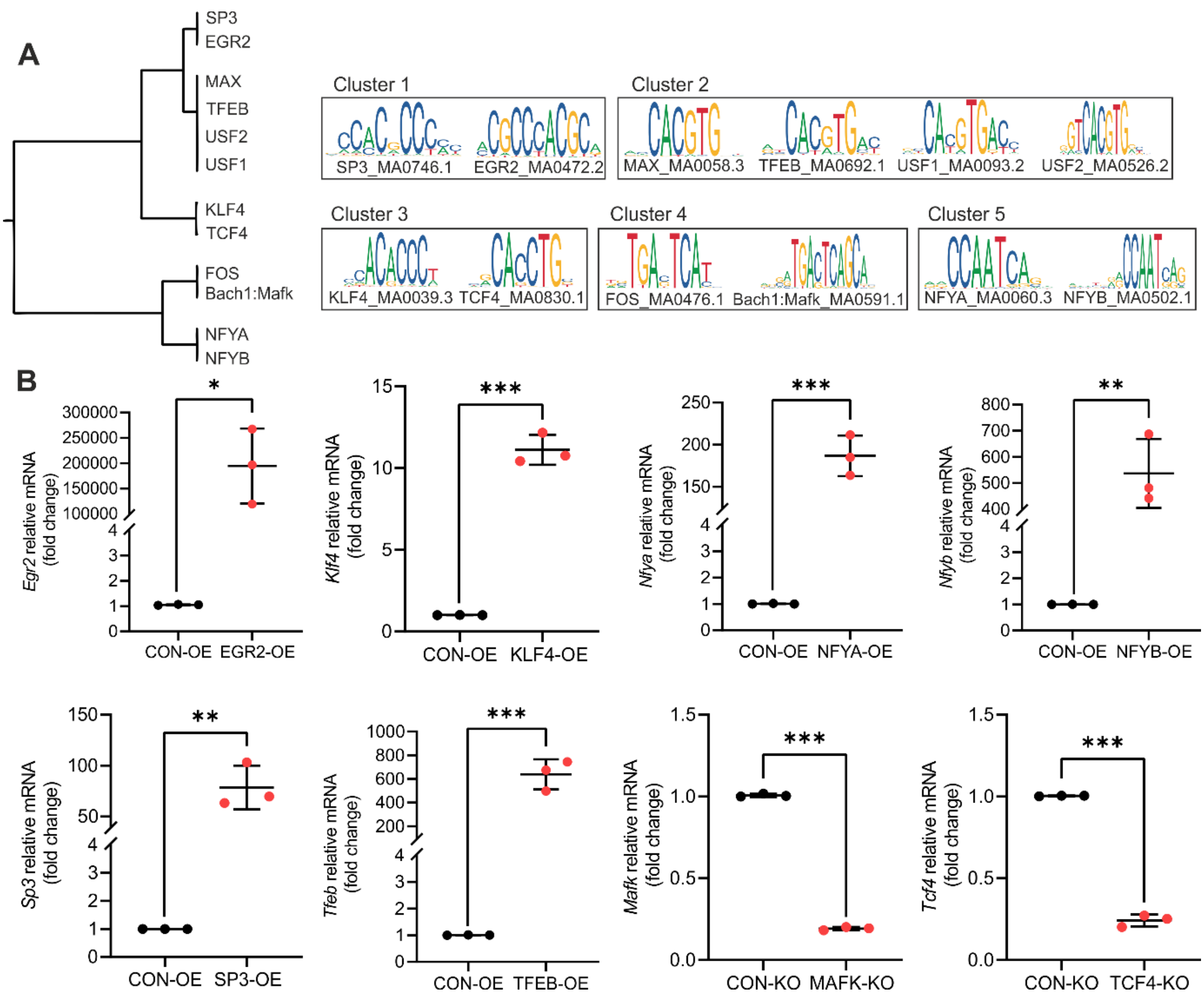

Figure S2. Lipotoxicity-sensitive transcription factors. (A) Transcription factor clustering analysis based on motif consensus sequence (left panel), with the motif logos comprised in the different clusters (right panel). (B) Transcript level of candidate transcription factors following overexpression (OE) or knockout (KO) in mouse hepatocytes compared to their corresponding controls (CON; n=3 independent experiments; values are mean ± SD; *p < 0.05, **p < 0.01 and ***p < 0.001).

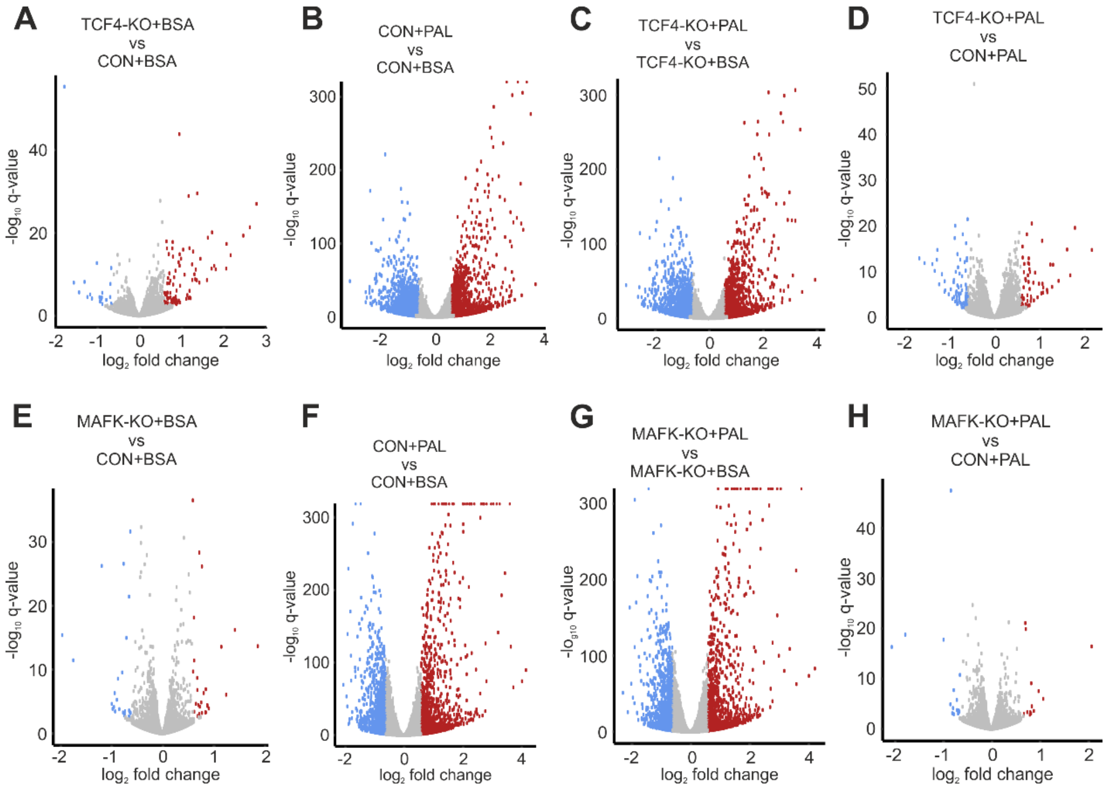

Figure S3. Transcriptomic analysis of MAFK and TCF4 knockout cells. (A-H) Volcano plots showing DEG under basal conditions and following PAL stimulation for 24 h in (A-D) TCF4-KO or (E-H) MAFK-KO mouse hepatocytes (n=3 per group; CON: control; blue and red dots denote significantly down- and up-regulated genes, respectively).

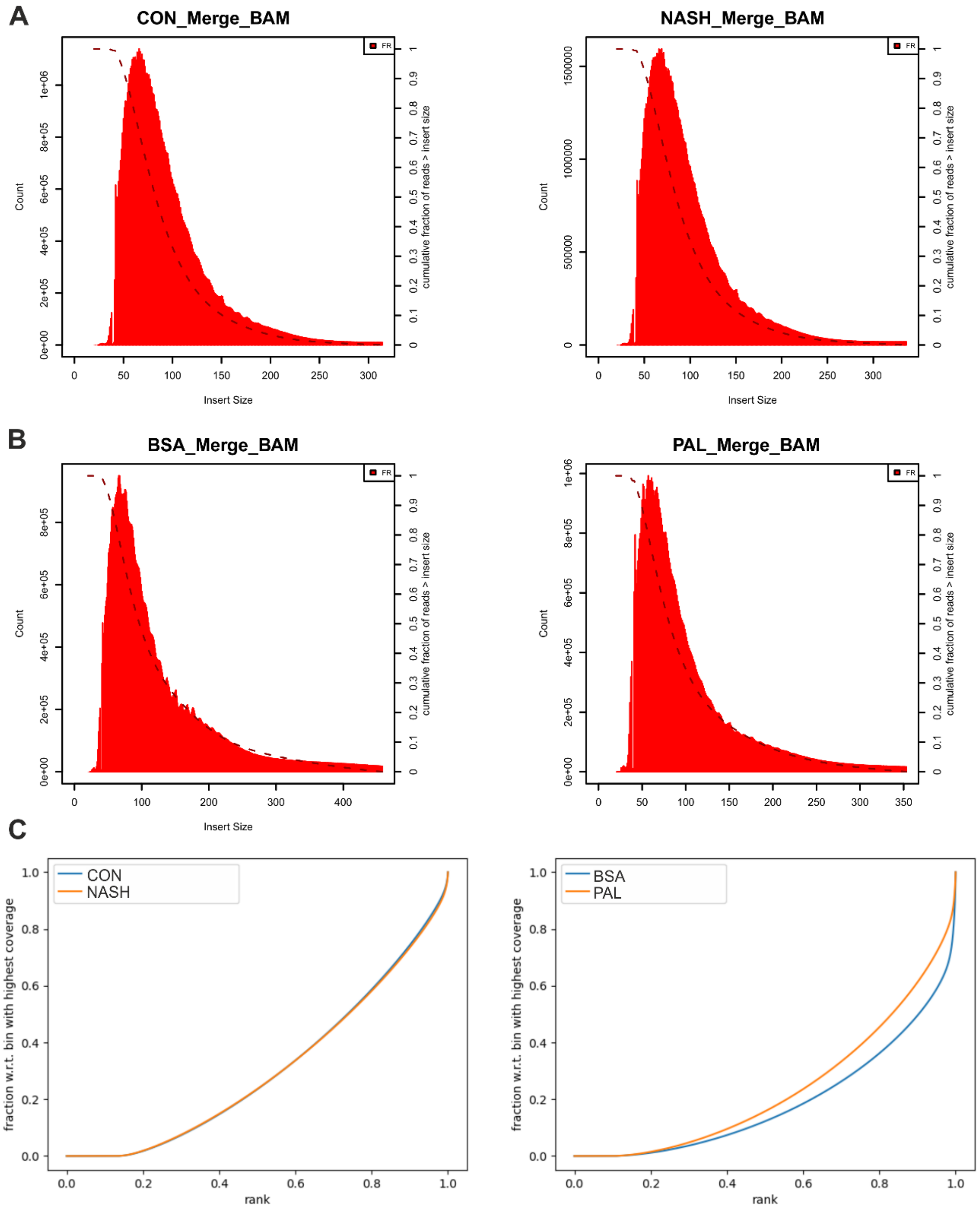

Figure S4. ATAC-seq quality control. (A and B) Fragment size distribution and (C) plotFingerprint analysis of ATAC-seq data.
